# Money matters: a multicenter cross-sectional study of depressive symptoms among the caregivers of children on peritoneal dialysis in Mainland China

**DOI:** 10.1186/s12882-020-02147-3

**Published:** 2020-11-10

**Authors:** Rui Zhao, Ying Gu, Xia Shen, Xianying Mai, Cheng Zhou, Yufen Zhang, Yihui Zhai, Qian Shen, Hong Xu, Qing Zhou

**Affiliations:** 1 Nephrology Department, Children’s Hospital of Fudan University, Shanghai, 201102 China; 2 Nursing Department, Children’s Hospital of Fudan University, Shanghai, China; 3 Nephrology Department, Children’s Hospital of Chongqing Medical University, Chongqing, China; 4 Nephrology Department, The 2nd Affiliated Hospital and Yuying Children’s Hospital of Wenzhou Medical University, Wen Zhou, Zhejiang Province China; 5 Nephrology Department, Zhengzhou Children’s Hospital, Zhengzhou, Henan Province China

**Keywords:** Cross-sectional studies, Caregivers, Child, Depression, Peritoneal dialysis

## Abstract

**Background:**

The caregivers of children on peritoneal dialysis face heavy care burdens and may have a high risk of depression. This study aimed to describe the prevalence and severity of depression and identify its related demographic and socioeconomic factors in the caregivers of children on peritoneal dialysis in China.

**Methods:**

A multicenter cross-sectional study was conducted in four pediatric dialysis centers in four tertiary children’s hospitals in mainland China. Primary caregivers of children with end-stage kidney disease and currently on peritoneal dialysis were screened and recruited from December 2018 to July 2019. A self-developed questionnaire and the Self-Rating Depression Scale (SDS) were administered to the participants by a trained nurse in each center. The chi-square test or Fisher’s exact test, one-way ANOVA, and the Mann-Whitney U test were used to compare the prevalence of depressive symptoms by demographic features. A multivariate logistic regression analysis was used to identify factors related to depressive symptoms in caregivers of children on peritoneal dialysis.

**Results:**

One hundred twenty-one caregivers were included in the data analysis. The mean age of the caregivers was 40.1 ± 8.1 years. More than 75% of the participants were female, married, and unemployed. The overall prevalence of depressive symptoms was 59%. In total, 46 (38%), 20 (17%) and 5 (4%) caregivers reported mild, moderate, and severe depressive symptoms, respectively. In the univariate analysis, caregivers with an average household income per month under 4000 RMB and caregivers of children undergoing laparoscopic surgery had a higher prevalence of depressive symptoms. Characteristics such as treatment center, duration on PD, PD modalities, and history of peritonitis episodes showed no difference in terms of the prevalence of depressive symptoms. The multivariate logistic regression analysis demonstrated that an average household income per month under 4000 RMB was the associated factor for caregivers’ depressive symptoms.

**Conclusions:**

The caregivers of children on peritoneal dialysis in mainland China were socially vulnerable and experienced depression. Those who had a higher average household income were less vulnerable to depression.

## Background

Kidney replacement therapy (KRT), including peritoneal dialysis (PD), hemodialysis, and kidney transplantation, is a life-saving treatment for children with end-stage kidney disease (ESKD). PD is recommended as the initial dialysis treatment of choice for ESKD in children aged 2 years or older and as the best option for infants [1, 2]. There are marked variations in the incidence or prevalence of ESKD in the pediatric population across countries, and the rate of accessible pediatric KRT varies with healthcare resources [1]. Around the world, the median incidence of KRT in children aged less than 20 years is approximately 9 per million age-related population [3]. In mainland China, from January 2007 to December 2011, 474 children received KRT, of which 177 (37.3%) underwent PD. Of the children on PD, 50.3% received automatic PD (APD) for their daily treatment [4]. With the progress made in PD surgery and care in recent years, the number of children on PD has increased. According to the International Pediatric Peritoneal Dialysis Network-China (IPDN-China) [5], by the end of August 2019, there were 470 PD children registered for and 258 currently on PD from 40 PD centers in China [6].

Caregiving, which is accompanied by objective stressors, including patients’ physical disabilities, cognitive impairment, and problem behaviors, has physical and mental health effects on caregivers [7], and depression is the most commonly reported outcome in caregivers of children with chronic conditions [8]. The prevalent depressive symptoms of caregivers impact not only the wellbeing of parents themselves but also the children’s wellbeing and family system. A systematic review of childhood cancer found that parental depression was associated with a higher level of children’s distress and symptoms and negatively impacted children’s school functioning [9]. In pediatric transplant children, caregivers’ depressive symptoms were related to children’s nonadherence and possibly increased rates of rejection [10]. In the pediatric care setting, the latest guideline proposed that high-quality PD descriptions should be designated to meet the mental needs of the family [11]. Thus, the mental distress of caregivers of children on PD merits more attention.

It is evident that depressive episodes often develop after a major life event and could persist with long-term stress. With dual parental and medical responsibilities, the parenting stress of caregivers of children with chronic disease has been associated with poorer psychological adjustment in caregivers [12, 13]. Compared with caregivers of a healthy child, caregivers of a child with PD have a higher chance of emotional distress [14]. Among caregivers of children on PD, dialysis for the management of kidney failure represents burdensome ongoing interventions and requires intense physical, mental, and financial care from caregivers [15]. In addition to managing medical treatment, routine follow-ups, and dietary requirements, PD caregivers have the added responsibilities of acquiring PD supplies, running cyclers or changing fluids, performing exit care, closely monitoring the children’s fluid balance, preventing PD-related infections, and preparing for future kidney transplantation. These responsibilities may predispose caregivers of children on PD to depression.

Despite the risk of depression, the psychological status of these caregivers of children on PD remains unknown and underinvestigated. This study aimed to understand the features of the demographics of caregivers of a child on PD in mainland China, to describe the prevalence and severity of depressive symptoms in the caregivers, and to identify related demographic and socioeconomic factors of depressive symptoms in the caregivers.

## Methods

### Aim

This study aimed to understand the features of demographics of caregivers with a child on PD, to describe the prevalence and severity of depression, and to identify its related demographic and socioeconomic factors in the caregivers of children on PD in mainland China.

### Study design and setting

A cross-sectional study on caregivers of children on PD was conducted from December 2018 to July 2019. The number of participants in four centers during the study period determined the sample size. A trained nurse in each center recruited participants from departments in four tertiary children’s hospitals in mainland China during the children’s clinic visits or upon admission to an inpatient ward. The four pediatric dialysis centers that were included had the most current pediatric dialysis cases registered with IPDN-China. These four hospitals include the Children’s Hospital of Fudan University, Shanghai; The 2nd Affiliated Hospital and Yuying Children’s Hospital of Wenzhou Medical University, Wen Zhou, Zhejiang Province; Children’s Hospital of Chongqing Medical University, Chongqing; and Zhengzhou Children’s Hospital, Zhengzhou, Henan Province. These centers had all launched PD training for caregivers. Each hospital follows up children in either an outpatient ward or an inpatient ward.

### Participants

#### Eligibility criteria

Caregivers with children under the age of 18 years and currently on PD.

##### Inclusion/exclusion criteria

The children had a diagnosis of ESKD [1] and had been on PD for at least 1 month. The caregivers of children on PD were those who performed PD daily care. If more than one caregiver is involved, only the primary caregiver will be included. The primary caregiver refers to the caregiver who performs daily dialysis and is responsible for the child’s daily treatment. The primary caregivers were also able to understand and communicate in Mandarin or another understandable dialect. The caregivers signed written informed consent to indicate their willingness to participate in the study. Those who failed to communicate with the trained nurse or had mental diseases or children who performed their daily care themselves were excluded from the study.

### Instruments

#### Demographic and treatment information of families with children on maintenance PD

A self-developed questionnaire was administered to collect the demographic and treatment information of the caregivers and children. For caregivers, the demographic information included their age, sex, residence, number of children cared for, relationship to the child, educational background, marital status, working status, average household income per month, and medical payment for PD. For the children on PD, the data collected included their age, sex, PD catheter placement, treatment modality, duration on PD, and history of peritonitis episodes.

#### Depressive symptoms

We used the Chinese version of the Self-Rating Depression Scale (SDS) to assess the severity of the caregivers’ depressive symptoms in the past week. The scale has been tested and validated among adults in China [16–18]. The instrument has 20 items, which are rated on a Likert scale ranging from 1 ~ 4 (never or very few to most of the time or all of the time). The average of the summed results multiplied by 1.25 was calculated to derive a standard score. The cutoff score was 50, with 50 ~ 59, 60 ~ 69, and 70 and above indicating mild, moderate, and severe depressive symptoms, respectively. The internal consistency of the Chinses version of SDS among caregivers was 0.92 [18]. In our study, a cutoff score of 50 was used to indicate the occurrence of depressive symptoms.

### Data collection

The data were collected in either the inpatient ward or the outpatient department. To reduce interrater bias, the PI (corresponding author) trained one nurse as a data collector in each center. The trained nurse in each center was responsible for screening participants, explaining the study, acquiring informed consent, and instructing caregivers on how to complete the questionnaire. After a caregiver had completed a questionnaire, the trained nurse in each center checked the questionnaire for completeness. The treatment information, including PD catheter placement, duration on PD, and previous peritonitis episodes, was verified by the trained nurse according to the medical records. For caregivers who experienced difficulty completing the questionnaire, such as those who were unable to read or write, the trained nurse read the questions and answers one by one and completed the questionnaire according to the participants’ responses. All the questionnaires were emailed to the PI. The study was approved by the Ethics Board of the Children’s Hospital of Fudan University (2018–299).

### Statistics

Two researchers independently entered all the information in EXCEL 2011 (Microsoft, Redmond, WA). All data were checked for logic errors and coded. We then performed all the statistical analyses in SPSS21.0 (IBM Corp. Armonk, NY). *P* values < 0.05 were considered statistically significant. A P-P plot was used to explore the normality of the data. For continuous variables such as age, duration on PD, mean and standard deviation were used to describe normally distributed data, and median and interquartile range were used to describe data with a skewed distribution. The chi-square test or Fisher’s exact test (categorical variables), one-way ANOVA (continuous variables with normal distribution), and the Mann-Whitney U test (continuous variables with skewed distribution) were used to compare the prevalence of depressive symptoms by demographic features. Odds ratios were calculated using the chi-square test or logistic regression in the case of categorical variables with more than two levels, such as treatment centers. Finally, the multivariate logistic regression was used to identify factors related to depressive symptoms in caregivers of children on PD. Factors related to the prevalence of depressive symptoms in the unadjusted analyses were included in the multivariate logistic regression. Meanwhile, factors considered to be clinically relevant, including caregivers’ age, average household income per month, residence, and duration on PD, were also included in the multivariate analysis. In addition, the linearity assumption between the logarithm of continuous variables such as caregivers’ age and the prevalence of depressive symptoms was tested using the Box-Tidwell test. Collinearity diagnostics were also performed to detect multicollinearity.

## Results

### Demographic information of the PD children and their primary caregivers

**Fig. 1:**
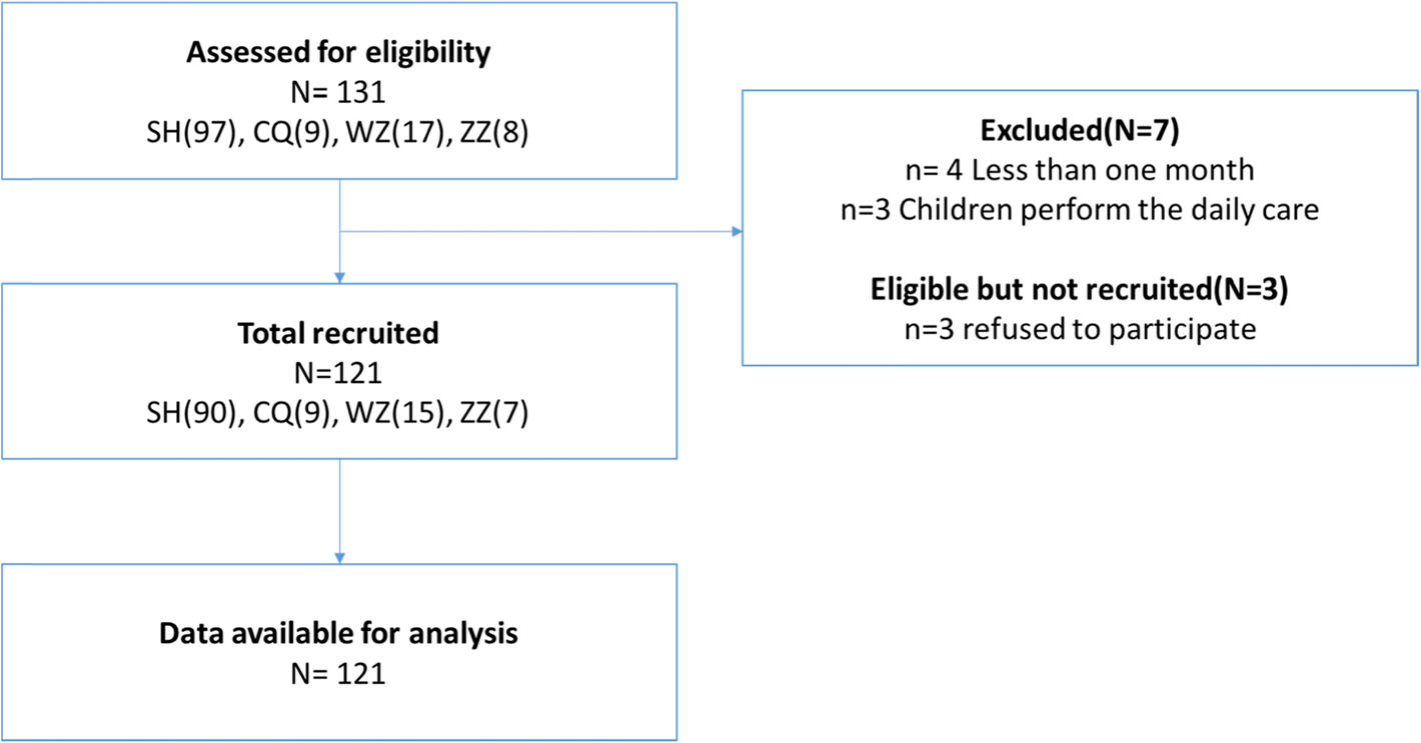
Recruitment flow diagram for children on PD and their caregivers. * SH, Shanghai center; CQ, Chongqing center; WZ, Wenzhou center; ZZ, Zhengzhou center

In all, 121 participants returned the questionnaire and were included in the data analysis (see Fig. 1). Overall, the mean age of the caregivers was 40.1 ± 8.1 years. Most caregivers were the children’s parents (91%), female (83%), married (93%), currently not working (77%), and with an average household income under 4000 RMB (78%). In total, 65 (62%) and 77 (64%) of them had a middle school degree or less and cared for more than one child. The mean age of their children was 10.5 ± 4.3 years. The median duration on PD was 11 months, with a minimum of 1 month and a maximum of 84 months. The demographic information of the children on PD and their caregivers from four centers is presented in Table 1.

**Table 1:** Characteristics of Familes with Children on PD

Characteristics	Overall (*n* = 121) No. (percentage)
Centers	
*Shanghai*	90 (74)
*Chongqing*	9 (7)
*Wenzhou*	15 (12)
*Zhengzhou*	7 (6)
Parents	110 (91)
Female (Caregiver’s sex)	101 (83)
Caregiver’s age (years old) ^a^	40.1 ± 8.1
Rural	58 (48)
Married	113 (93)
Middle school and below	75 (62)
Not working	93 (77)
Caring for more than one child	77 (64)
Medical insurance	71 (59)
Average household income per month < 4000 RMB	94 (78)
Male (Child’s sex)	71 (59)
Child’s age (years old) ^a^	10.5 ± 4.3
Laparoscopic surgery	83 (69)
APD	93 (77)
Duration on PD (months) ^b^	11 (3, 24)
Peritonitis episodes history	23 (19)

^a.^Mean ± SD,

^b.^Median (25th percetile, 75th percentile)

*PD peritoneal dialysis, *APD* automatic peritoneal dialysis

### Prevalence and severity of depressive symptoms

The overall prevalence of depressive symptoms was 59% (95% CI [49.8, 67.6%]). In total, 46 (38%), 20 (17%) and 5 (4%) caregivers reported mild, moderate, and severe depressive symptoms, respectively. There were no significant differences between the depression group and the nondepression group concerning child’s age (10.0 ± 4.1 years vs 9.6 ± 4.6 years, F = 0.1, *P* = 0.7), caregiver’s age (38.3 ± 8.6 years vs 41.1 ± 8.4 years, F = 2.2, *P* = 0.1), and duration on PD (9 months vs 10 months, Z = -0.5, *P* = 0.6). Variables including treatment center, child’s sex, PD modalities, history of peritonitis episodes, caregivers, caregiver’s sex, educational background, marital status, working status, caring for more than one child, and hospital payment did not show an influence on the prevalence of depressive symptoms. Caregivers with an average household income per month < 4000 RMB (OR = 3.9 95% CI [1.6, 9.6], *P* = 0.002) and those with children undergoing laparoscopic surgery (OR = 2.3 95% CI [1.1, 5.0], *P* = 0.04) had a higher prevalence of depressive symptoms (see Table 2).

**Table 2:** Unadjusted Analyses of the Prevalence of Depressive Symptoms of Caregivers with Children on PD

	Prevalence No. (Percentage)	OR 95 Confidence Interval
Centers		
*Shanghai*	56 (62)	Ref
*Chongqing*	5 (56)	0.8 (0.2 to 3.0)
*Wenzhou*	8 (53)	0.7 (0.2 to 2.1)
*Zhengzhou*	2 (29)	0.2 (0.0 to 1.3)
Child’s sex		
*Male*	41 (58)	0.9 (0.4 to 1.9)
*Female*	30 (60)	Ref
PD catheter placement		
*Laparoscopic surgery*	54 (65)	2.3 (1.1 to 5.0) ^a*^
*Open surgery*	17 (45)	Ref
PD modalities		
*APD*	58 (62)	1.9 (0.8 to 4.5)
*CAPD*	13 (46)	Ref
History of peritonitis episodes		
*Yes*	14 (61)	1.1 (0.4 to 2.8)
*No*	57 (58)	Ref
Caregivers		
*Parents*	65 (59)	1.2 (0.3 to 4.2)
*Grandparents*	6 (55)	Ref
Caregiver’s sex		
*Male*	10 (50)	0.7 (0.3 to 1.7)
*Female*	61 (60)	Ref
Residence		
*Rural*	38 (66)	1.7 (0.8 to 3.6)
*Urban*	33 (52)	Ref
Marital status		
*Married*	66 (58)	0.8 (0.2 to 3.7)
*Widowed or divorced*	5 (63)	Ref
Educational Background		
*Middle school and below*	48 (64)	1.8 (0.8 to 3.8)
*High school and above*	23 (50)	Ref
Work status		
*Working*	14 (50)	0.6 (0.3 to 1.5)
*Not working*	57 (61)	Ref
Caring for more than one child		
*Yes*	48 (62)	1.5 (0.7 to 3.2)
*No*	23 (52)	Ref
Hospital payment		
*Medical insurance*	43 (61)	1.2 (0.6 to 2.5)
*Out of pocket*	28 (56)	Ref
Average household income per month		
*< 4000 RMB*	62 (66)	3.9 (1.6 to 9.6) ^a**^
*≥ 4000 RMB*	9 (33)	Ref

^a.^*P* < 0.05,

*0.01 < *P <* 0.05, ** *P* < 0.01

*Ref* reference

### Factors associated with the prevalence of depressive symptoms

The multivariate logistic regression was used to explore factors related to depressive symptoms in caregivers of children on PD. Variables including PD catheter replacement, average household income per month, residence, duration on PD, and caregiver’s age were included in the model. The significance test of model coefficients was χ^2^ = 15.6, *P* = 0.008. The results showed that an average household income per month under 4000 RMB was associated with caregiver’s depressive symptoms (OR = 2.8, 95% CI [1.1, 7.5], *P* = 0.04), as shown in Table 3.

**Table 3:** Multivariate Logistic Regression of the Prevalence of Depressive Symptoms of Caregivers with Children on PD

Variables	Exp (B)	95%CI for Exp (B)	Sig.
Average household income per month			
*< 4000 RMB*	2.8	1.1 to 7.5	0.04*
*≥ 4000 RMB*	Ref		
Residency			
*Rural*	1.4	0.7 to 3.2	0.4
*Urban*	Ref		
PD catheter placement			
*Laparoscopic surgery*	1.9	0.7 to 4.8	0.2
*Open surgery*	Ref		
Caregiver’s age (years)	0.95	0.91 to 1.00	0.06
Duration on PD (months)	1.01	0.98 to 1.04	0.4

*Ref* reference, *PD* peritoneal dialysis

*P < 0.05;

## Discussion

Family-oriented outcomes and measures for chronic pediatric diseases have been well studied in diseases such as cerebral palsy, cystic fibrosis, and diabetes [19]. The factors and consequences of parental distress, including depression, have been studied in childhood diseases such as cancer [9] and injuries. In contrast, very few reports have investigated these factors and consequences among children on PD due to the relatively low prevalence of ESKD and KRT. Our study tried to understand the status quo of caregivers of children on PD in mainland China regarding their demographics, overall depressive symptom prevalence, and related factors of depressive symptoms.

Our study found that primary caregivers of children on PD in mainland China were mostly children’s parents in their forties and were female, as in other studies on Chinese caregivers of children with other chronic diseases [20]. Other demographic features of the caregivers of children on PD in China include being married, currently not working, having middle school degrees or below, caring for more than one child, and having an average household income under 4000 RMB. A low level of educational attainment [20], a low level of household income [21], and unemployment [22] were found in past studies to be associated with the prevalence of depressive symptoms. This might explain the relatively high prevalence of depressive symptoms in our study. Among caregivers of a child on PD, the overall prevalence of depressive symptoms was 59%, as measured by SDS, which tended to be higher than in some reports of caregivers of children with pediatric chronic diseases. The prevalence of depressive symptoms in mothers of a chronically ill child was 23.0% (130/566), as measured by the Hospital Anxiety and Depression Scale (HADS) [23]. Wang et al’s study showed that the prevalence of depressive symptoms of Chinese caregivers of children with adolescent idiopathic scoliosis was 14.1% (9/64), as measured by the Patients’ Health Questionnaire-9 (PHQ-9, 20]. However, due to the variation in assessment tools, the difference might be insignificant.

We also found that the number of participants varied greatly across centers. This may reflect the fact that the financial and health resources of care of pediatric PD have been unevenly distributed and more aggregated in capital cities and municipalities. To access these healthcare resources, some families have to travel far. Shanghai center, for instance, treated children from more than 20 provinces, including children from Xinjiang. This background information may offer more understanding of the mental distress of caregivers of children on PD in mainland China. Additionally, although possible inconsistency in PD care in the included treatment centers may exist, it is hardly detectable with the small number of participants in some centers.

The multivariate logistic regression showed that the prevalence of depressive symptoms was not associated with children’s PD-related features. Research in other pediatric chronic conditions also had similar findings. According to van Oers et al’s cross-sectional study, parents’ depressive symptoms were strongly associated with practical problems in daily life and parenting stress but not with illness-related characteristics of the children [23]. Furthermore, in Cousino’s study, parental stress was associated with greater parental responsibility for treatment management and was unrelated to illness duration and severity across illness populations [13]. In our study, the prevalence of depressive symptoms was related to the caregivers’ demographic factors, mainly income. We observed that a low household income per month (below 4000 RMB), compared with a high household income per month (above 4000 RMB), increased the prevalence of depression. Similar findings were shown in caregivers of children on hemodialysis, for whom financial and bureaucratic problems were the most common stress factors [24]. Other empirical studies on the caregivers of children on hemodialysis have substantiated these findings. Sezer’s study on maintenance hemodialysis patients and their caregivers found that monthly income was negatively correlated with the Beck Depression Inventory [25].

The heavy financial burden of the caregivers could be explained by life-long treatment with persistent financial challenges, limited insurance coverage, and difficulty in accessing specialized pediatric resources. Based on data from the China Health Insurance Research Database and the Commercial Health Insurance Database [26], in 2015, the median annual overall cost per adult for PD was 73,266 RMB. Basic health insurance covered 76.6% of the total medical expenditure for dialysis patients (including hemodialysis). With a real GDP per capita of 50,237 in 2015 in China [27], approximately 34% of an individual’s income was spent on dialysis treatment. However, these data may not fully uncover the financial difficulties of children on PD. First, the proportions of reimbursement and insurance type vary by province for children. Next, cyclers and compatible PD cassettes are the prerequisites for APD. One piece of PD cassette costs caregivers 42 RMB per day, and these, together with APD supplies such as iodine caps and exit-care products, are not reimbursed by basic health insurance. Additionally, similar to the fact that specialized pediatric resources are located in large cities, PD fluids and cassettes for APD are not available in the capital cities of every province, which increases expenditures on delivery or travel.

### Limitations

First, our samples were from relatively well-organized pediatric dialysis centers in China, while those that failed to report their pediatric PD cases on IPDN may not be represented in the sample. Thus, the findings in our study may not be generalizable to those cases. Second, to assist illiterate or older caregivers in reporting, nurses helped 6 caregivers complete the questionnaire after reading the questionnaire verbatim. Thus, there were chances that inconsistencies might occur with the assisted reports. Third, the related factors analyzed in the study were not exhaustive, and psychological characteristics, such as social support, self-efficacy, or coping style, were not included in the analysis. Fourth, due to the use of a variety of tools to assess depression, the prevalence of depressive symptoms among caregivers of children with different medical conditions might not be comparable.

### Implications for practice and research

The mental wellbeing of caregivers of children on PD warrants more attention, and substantial financial assistance could be a possible way to reduce the prevalence of depressive symptoms. Future studies should focus on understanding the relationship between mental welling and the financial difficulties of families with children on PD. Longitudinal studies would provide better evidence to understand the change in caregivers’ mental health and its influence on children’s health-related outcomes, PD-related outcomes, and family systems across the PD trajectory.

## Conclusions

In conclusion, the caregivers of children on PD in mainland China were socially vulnerable and experienced a high prevalence of depressive symptoms. Among the demographic and treatment factors of families with a child on PD in mainland China, average household income was an associated factor for the prevalence of depressive symptoms.

## Data Availability

The datasets used and/or analyzed during the current study are available from the corresponding author on reasonable request.

## Abbreviations

APD: Automatic peritoneal dialysis
ESKD: End-stage kidney disease
GDP: Gross domestic product
IPDN: International Peritoneal Dialysis Network
PD: Peritoneal dialysis
PI: Primary investigator
KRT: Kidney replacement therapy
SDS: The Self-Rating Depression Scale

## References

[1] Kidney Disease: Improving Global Outcomes (KDIGO) CKD Work Group: KDIGO 2012 Clinical practice guideline for the evaluation and Management of Chronic Kidney Disease. Kidney Int Suppl. 2013;3(1):1–150.

[2] National Institute for Health and Care Excellence: Chronic kidney disease (stage 5): peritoneal dialysis [EB/OL]. (2011.07.27)[2020.03.30]. http://nice.org.uk/guidance/cg125. 22536622

[3] Harambat J, van Stralen KJ, Kim JJ, Tizard EJ (2012). Epidemiology of chronic kidney disease in children. Pediatr Nephrol.

[4] Working Group for National Survey on Status of Diagnosis and Treatment of Childhood Renal Diseases: Survey of renal replacement therapy in childhood with chronic renal failure. Zhonghua Er Ke Za Zhi. 2013;51(7):491–4. 24267128

[5] Zhai Y, Liu X, Yang Q, Dang X, Sun S, Shao X, Liu X, Wu Y, Bai H, Mao J, et al. IPDN-China promotes the development of pediatric dialysis in China. Pediatr Nephrol. 2020. 10.1007/s00467-020-04630-3. 10.1007/s00467-020-04630-3 32529322

[6] International Peritoneal Dialysis Network China. general statistics [EB/OL]. (2019.08.23)[2020.02.26]. http://103.37.159.89/statistics/stats_general.php.

[7] Schulz R, Sherwood PR (2008). Physical and mental health effects of family caregiving. Am J Nurs.

[8] Law E, Fisher E, Eccleston C, Palermo TM (2019). Psychological interventions for parents of children and adolescents with chronic illness. Cochrane Database Syst Rev.

[9] Sultan S, Leclair T, Rondeau E, Burns W, Abate C (2016). A systematic review on factors and consequences of parental distress as related to childhood cancer. Eur J Cancer Care (Engl).

[10] Annunziato RA, Stuber ML, Supelana CJ, Dunphy C, Anand R, Erinjeri J, Alonso EM, Mazariegos GV, Venick RS, Bucuvalas J (2020). The impact of caregiver post-traumatic stress and depressive symptoms on pediatric transplant outcomes. Pediatr Transplant.

[11] Brown EA, Blake PG, Boudville N, Davies S, de Arteaga J, Dong J, Finkelstein F, Foo M, Hurst H, Johnson DW (2020). International Society for Peritoneal Dialysis practice recommendations: prescribing high-quality goal-directed peritoneal dialysis. Perit Dial Int.

[12] Wood BL, Brown ES, Lehman HK, Khan DA, Lee MJ, Miller BD (2018). The effects of caregiver depression on childhood asthma: pathways and mechanisms. Ann Allergy Asthma Immunol.

[13] Cousino MK, Hazen RA (2013). Parenting stress among caregivers of children with chronic illness: a systematic review. J Pediatr Psychol.

[14] Tsai TC, Liu SI, Tsai JD, Chou LH (2006). Psychosocial effects on caregivers for children on chronic peritoneal dialysis. Kidney Int.

[15] Gilbertson EL, Krishnasamy R, Foote C, Kennard AL, Jardine MJ, Gray NA (2019). Burden of care and quality of life among caregivers for adults receiving maintenance Dialysis: a systematic review. Am J Kidney Dis.

[16] Lee HC, Chiu HF, Wing YK, Leung CM, Kwong PK, Chung DW (1994). The Zung self-rating depression scale: screening for depression among the Hong Kong Chinese elderly. J Geriatr Psychiatry Neurol.

[17] Peng H, Zhang YJ (2013). Y G: analysis of reliability and validity of Chinese version SDS scale in women of rural area. Chin J Shanghai Med.

[18] Qu G, Wang L, Tang X, Wu W, Zhang J, Sun Y (2020). Association between caregivers' anxiety and depression symptoms and feeding difficulties of preschool children: a cross-sectional study in rural China. Arch Pediatr.

[19] Khangura SD, Karaceper MD, Trakadis Y, Mitchell JJ, Chakraborty P, Tingley K, Coyle D, Grosse SD, Kronick JB, Laberge AM (2015). Scoping review of patient- and family-oriented outcomes and measures for chronic pediatric disease. BMC Pediatr.

[20] Wang H, Li T, Yuan W, Zhang Z, Wei J, Qiu G, Shen J (2019). Mental health of patients with adolescent idiopathic scoliosis and their parents in China: a cross-sectional survey. BMC Psychiatry.

[21] Chen Y, Bennett D, Clarke R, Guo Y, Yu C, Bian Z, Ma L, Huang Y, Sun Q, Zhang N (2017). Patterns and correlates of major depression in Chinese adults: a cross-sectional study of 0.5 million men and women. Psychol Med.

[22] Liu Y, Fu R, Roberto KA, Savla J (2019). Depressive symptoms among adult children caregivers in China: moderating effects of working status and gender. Aging Ment Health.

[23] van Oers HA, Haverman L, Limperg PF, van Dijk-Lokkart EM, Maurice-Stam H, Grootenhuis MA (2014). Anxiety and depression in mothers and fathers of a chronically ill child. Matern Child Health J.

[24] Cimete G (2002). Stress factors and coping strategies of parents with children treated by hemodialysis: a qualitative study. J Pediatr Nurs.

[25] Sezer S, Uyar ME, Bal Z, Tutal E, Ozdemir Acar FN (2013). The influence of socioeconomic factors on depression in maintenance hemodialysis patients and their caregivers. Clin Nephrol.

[26] Wang F, Yang C, Long J, Zhao X, Tang W, Zhang D, Bai K, Su Z, Gao B, Chu H (2019). Executive summary for the 2015 Annual data report of the China kidney disease network (CK-NET). Kidney Int Suppl (2011).

[27] National Bureau of Statistics of China. Annual Data [EB/OL]. (2015)[2020–02]. http://data.stats.gov.cn/search.htm?s=2015%20%E4%BA%BA%E5%9D%87%E5%9B%BD%E5%86%85%E7%94%9F%E4%BA%A7%E6%80%BB%E5%80%BC.

